# Effect of Storage Time on DNA Obtained from Umbilical Cord Used in Forensic Identification

**DOI:** 10.3390/genes17091066

**Published:** 2026-09-03

**Authors:** Marta Ortega-Martínez, Devani Moreno-Sánchez, Jenifer Hernández-Martínez, María-de-Lourdes Chávez-Briones, Yareth Gopar-Cuevas, Jaime García-Juárez, Gilberto Jaramillo-Rangel

**Affiliations:** 1 Department of Pathology, School of Medicine, Autonomous University of Nuevo Leon, Monterrey 64460, Mexico; marta.ortegamrt@uanl.edu.mx (M.O.-M.); devanimorenosanchez@gmail.com (D.M.-S.); mtz.jenifer022@gmail.com (J.H.-M.); mdlourdes.chavezbrn@uanl.edu.mx (M.-d.-L.C.-B.); yareth.goparcu@uanl.edu.mx (Y.G.-C.); 2 Department of Histology, School of Medicine, Autonomous University of Nuevo Leon, Monterrey 64460, Mexico; jaime.garciajr@uanl.edu.mx

**Keywords:** umbilical cord, storage time, forensic identification

## Abstract

Background/Objectives: In the identification of human remains, their genetic profile can be compared with that obtained from biological samples of the person from whom they are suspected to have come. Our aim was to evaluate the effect of storage time on DNA obtained from umbilical cord that could be used as a reference in forensic identification. Methods: Fifteen umbilical cords stored by the donors in their homes for a period of between 0.5 and 47 years were collected. Saliva samples were obtained for use as a reference. DNA extraction was performed using the PrepFiler Express BTA™ Forensic DNA Extraction Kit and quantified using the Quantifiler™ HP DNA quantification kit. STR profiling was performed using the GlobalFiler™ PCR Amplification Kit. Alleles were detected using an ABI PRISM^®^ 3500 genetic analyzer. Results: In samples from 0.5 to 21 years of age, DNA concentration and degradation index (DI) did not follow a linear pattern over time; the genetic profiles obtained matched that of the corresponding saliva sample. Older samples showed lower DNA concentration values and higher DIs. The percentage of alleles recovered decreased to 31% in the oldest umbilical cord analyzed. However, from a sample stored for 31 years, a DNA concentration and DI similar to the younger samples were obtained, along with a complete STR profile. Conclusions: It is possible to obtain STR profiles useful for forensic identification from umbilical cords even after decades of storage; however, success is highly variable and cannot be predicted from storage time alone.

## 1. Introduction

In many cases of personal identification of human remains, a comparison is made between their genetic profile and that of members of their presumed family, usually the parents. However, this procedure does not necessarily provide conclusive proof of identity, as ambiguous results may occur due to the presence of mutations or false matches with people who are not related to the victim [1,2]. To resolve these difficulties, the genetic profile of the remains can be compared with that obtained from personal items or biological samples of the person from whom the remains are suspected to have originated [3,4,5].

In some countries, it is customary to preserve the umbilical cord of newborns. DNA obtained from this tissue could be used in some cases for the forensic identification of human remains. There are few reports in the literature on this subject, mainly isolated cases or case series [6,7,8,9,10], and the influence of storage time on the results obtained has not been systematically analyzed. Furthermore, most of those studies did not utilize the highly sensitive and specific technology employed today, such as systems allowing for the simultaneous detection of multiple genetic markers, and DNA quantification via real-time PCR, which can indicate the degree of sample degradation and the presence of PCR reaction inhibitors.

In this work, we evaluate the feasibility of STR profiling of umbilical cords stored up to 47 years.

## 2. Materials and Methods

**Figure 1:**
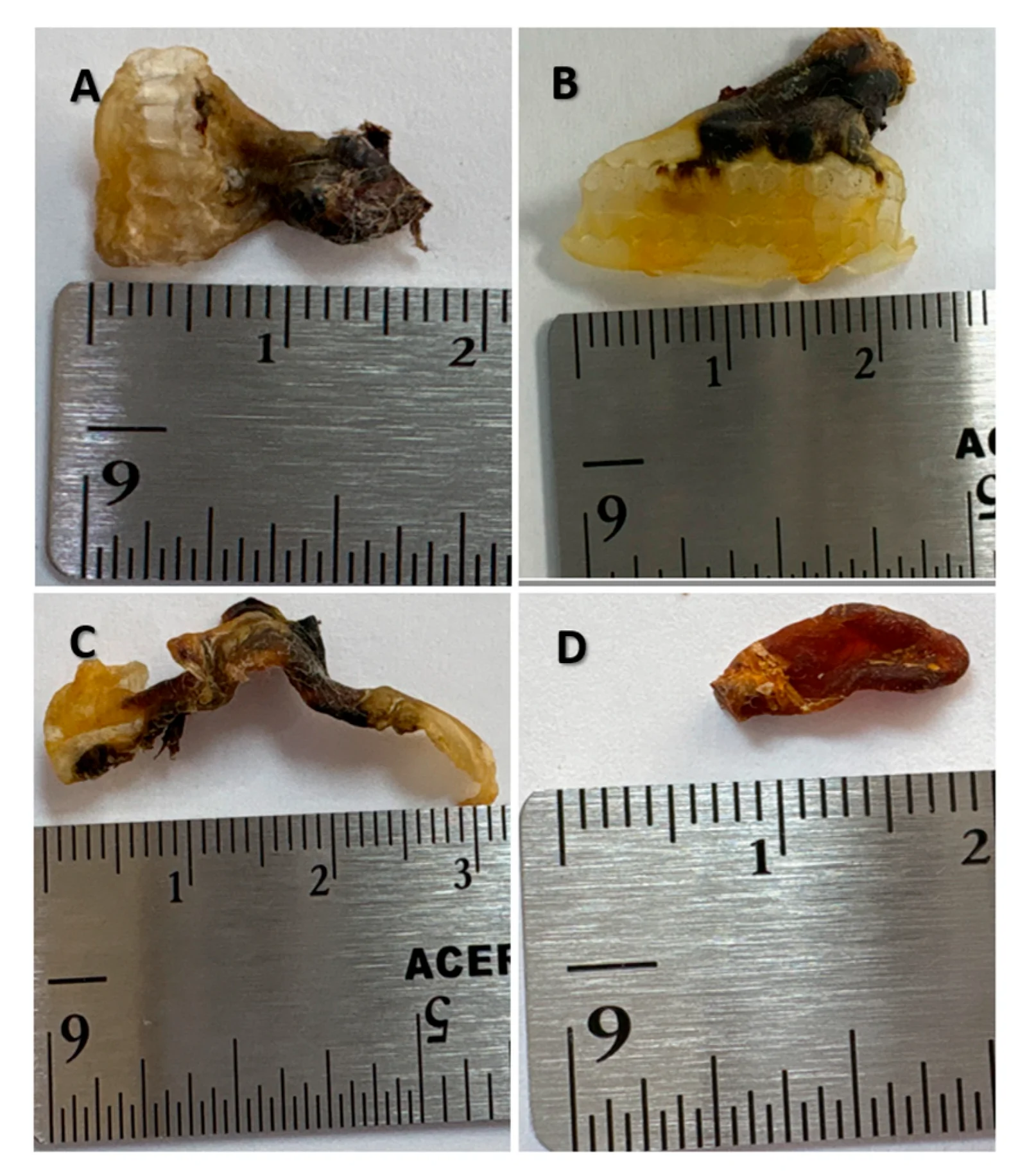
Examples of umbilical cords analyzed. Samples were stored for: (**A**) 2 years; (**B**) 19 years; (**C**) 31 years; (**D**) 47 years.

Fifteen umbilical cords stored for a period of between 0.5 and 47 years were collected (Figure 1). The samples were stored by the donors in their homes, at room temperature, in the containers shown in Table 1, in the city of Monterrey, Mexico (mean temperature: 23 °C, mean relative humidity: 66% [11,12]). DNA was isolated from an approximately 30 mg fragment of umbilical cord using the PrepFiler Express BTA™ Forensic DNA Extraction Kit (Applied Biosystems, Foster City, CA, USA). Briefly, samples were incubated overnight at 56 °C with gentle shaking in lysis buffer from the kit together with proteinase K (200 μg/mL) and 1 M DTT. Afterwards, they were centrifuged, and the supernatants were subjected to DNA extraction in the AutoMate™ Instrument (Applied Biosystems, Foster City, CA, USA) following the protocol provided by the manufacturer [13]. On the other hand, saliva samples were obtained from the donors of these tissues, and DNA was extracted from them using a Chelex protocol [14].

The DNA samples were quantified by real-time PCR on the 7500 ABI detection system using the Quantifiler™ HP DNA quantification kit following the manufacturer’s recommendations (Applied Biosystems, Foster City, CA, USA) [15]. The quantification kit also detects the presence of PCR inhibitors using an internal PCR control (IPC). PCR inhibition is assumed in a sample when its IPC cycle threshold (CT) shows a shift ≥ 2 cycles compared to the CT value of the non-template control (NTC). The kit also can determine the level of DNA degradation, expressed as a degradation index (DI), by dividing the concentration of a small amplicon (SA, 80 bp) by that of a large amplicon (LA, 214 bp). The CT values of the SAs and LAs were also considered in the analysis of the results. For results to be considered reliable, the slope of the standard curve should be in the range of −3.0 to −3.6 for the SA and −3.1 to −3.7 for the LA, and for both curves the correlation coefficient value (R^2^) should be equal to or greater than 0.99. The HID Real-Time PCR Analysis Software v1.3 (Applied Biosystems, Foster City, CA, USA) was used for data analysis.

DNA typing was carried out using the GlobalFiler™ PCR Amplification Kit in a ProFlex™ PCR Thermal Cycler. A DNA input of 1 ng was amplified according to the manufacturer’s protocols. For samples with low DNA concentration, the maximum permitted volume of undiluted DNA was added to the reaction mixture, in accordance with the kit’s amplification protocol (Applied Biosystems, Foster City, CA, USA) [16]. Amplified PCR products were electrophoretically separated on a capillary containing POP-4 polymer in an ABI PRISM^®^ 3500 genetic analyzer, and the GeneMapper^®^ IDX-v1.6 software was used for genotyping following the protocols provided by the manufacturer (Applied Biosystems, Foster City, CA, USA) [17,18]. For allele labeling, an analytical threshold of 55 relative fluorescence units (RFU) was used, while a stochastic threshold of 300 RFU was established for the designation of homozygotes. To calculate the percentage of profile completeness, the number of alleles obtained from each sample was compared with that of its corresponding saliva sample, which represented 100%.

## 3. Results

In order to analyze all the samples in the study, it was necessary to perform four quantification assays. The slopes of the standard curves were in the range of −3.0 to −3.1 for the SA, and of −3.2 to −3.3 for the LA, while the R^2^ value ranged between 0.995 and 1.0. Therefore, the results of DNA quantification assays can be considered reliable.

In umbilical cord samples from 0.5 to 21 years of age, DNA concentration values and DIs were variable and, in general, did not follow a linear pattern over time. In all samples, the genetic profile obtained matched that of the corresponding saliva sample for all alleles. With the exception of one sample, older samples showed lower DNA concentration values and higher DIs. The percentage of identical alleles between tissue and saliva samples decreased to 31% in the oldest umbilical cord analyzed (Table 1, Figures S1–S3 in Supplementary Materials).

Except for the samples stored for 12 and 21 years, the IPC CT values were within ±1 of the corresponding values for the NTCs (CT = 28–29). In the 26-year-old sample, it was not possible to obtain a CT value for the LA due to the failed amplification of the large target in the quantitative PCR. CT values for LA were high in one of the 31-year-old samples and in the 47-year-old sample. The CT value for SA was high only in the 26-year-old sample. CT values for small and large amplicons were normal in the remaining samples (Table 2).

**Figure 2:**
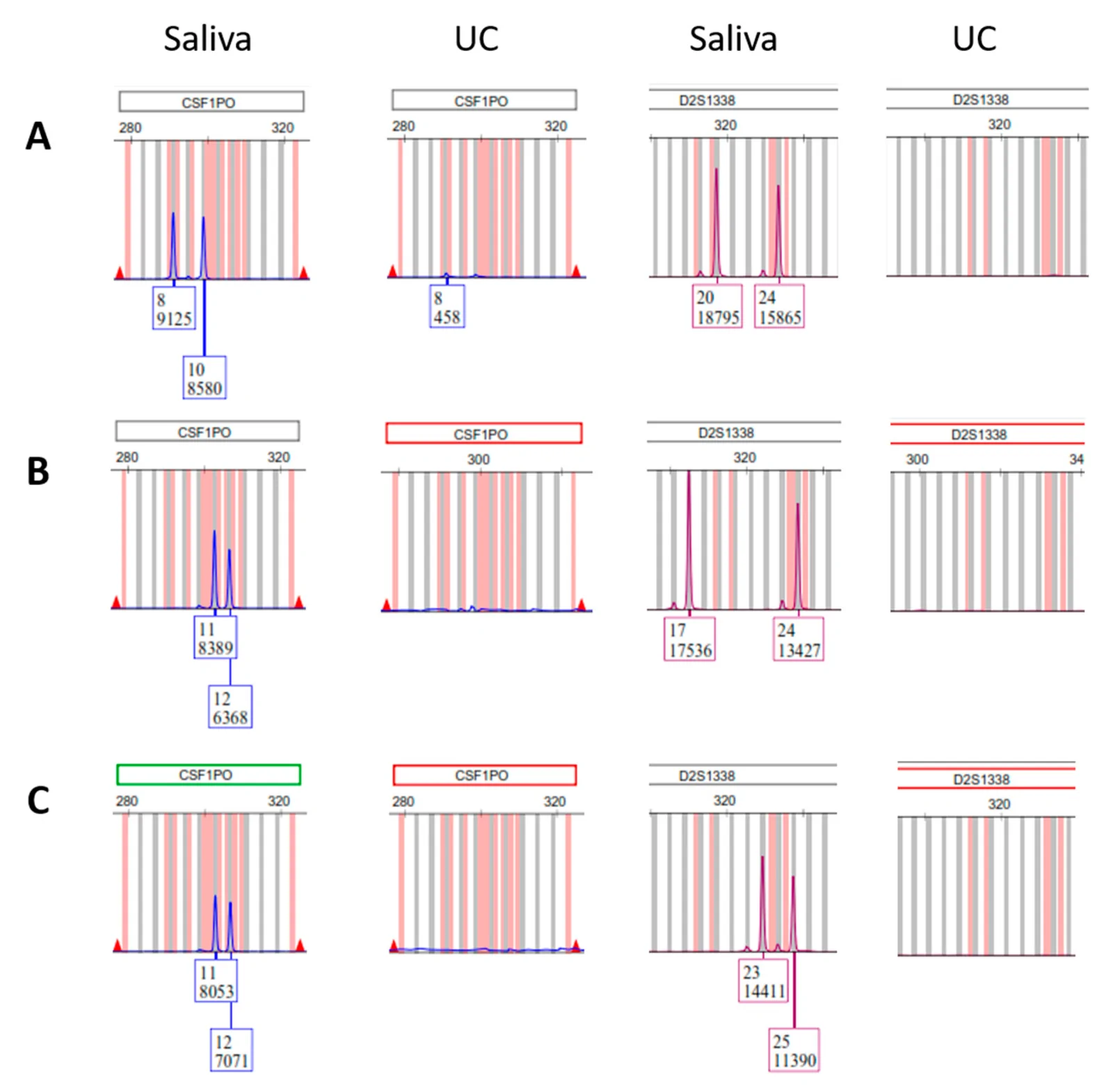
Fragments of electropherograms comparing peaks obtained from umbilical cord (UC) samples and saliva samples from their respective donors. (**A**) The 26-year-old UC showed complete loss of one allele at the locus CSF1PO, while the other allele of this locus showed a lower relative fluorescence units (RFU) value than that of its respective saliva sample. In addition, this UC sample had complete loss of both alleles at locus D2S1338. Both the 31-year-old UC (sample 31a) (**B**) and the 47-year-old UC (**C**) had complete loss of both alleles at loci CSF1PO and D2S1338. The labels show the allele calls at the top and the relative fluorescence units (RFU) at the bottom.

**Figure 3:**
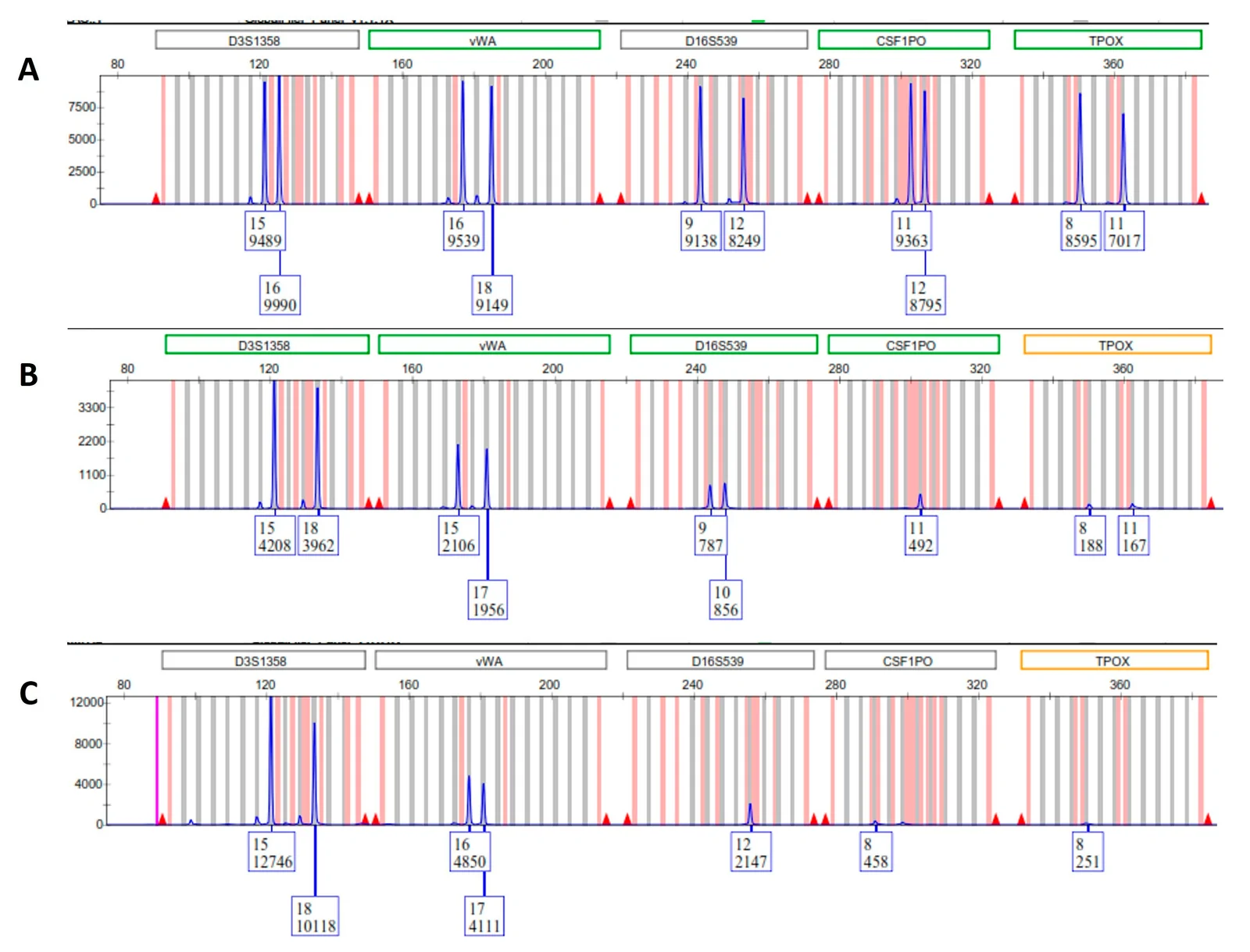
Ski slope effect. In degraded samples, DNA fragmentation can produce electropherograms showing high peaks for shortest loci (on the left side) and low peaks for longest loci (on the right side). (**A**) A sample that contained non-degraded DNA (sample stored for 2 years, DI 0.8) produced an electropherogram in which the peak heights of short and long loci were similar. In contrast, samples that contained more degraded DNA ((**B**), sample stored for 0.5 years, DI 5.3; (**C**), sample stored for 26 years, in which even the large target amplification was not achieved in quantitative PCR) produced lower amplification efficiency of long loci compared to short loci. The labels show the allele calls at the top and the relative fluorescence units (RFU) at the bottom. DI: degradation index.

Figure 2 shows fragments of electropherograms with examples of loci from umbilical cord samples that showed incomplete genetic profiles compared to their respective saliva samples (see also Table S1 in Supplementary Materials). With the exception of the sample stored for 2 years, the ski slope effect was observed, to a greater or lesser degree, in the electropherograms of all the others (Figure 3).

## 4. Discussion

In the process of identification of human remains through DNA profiling, the most reliable approach is to use biological samples from the individual presumed to be the source of the remains as reference material for comparative genetic analysis. One of those samples could be the umbilical cord. In this work, we analyzed the quantity, integrity and presence of PCR inhibitors in DNA obtained from umbilical cords with different storage times, as well as the usefulness in forensic identification of STR genetic profiles from these samples.

The samples were stored in the donors’ homes, at room temperature, and in the containers described in Table 1. There are official guidelines that establish the ideal conditions for the long-term storage of biological samples, so that they have the least possible degree of degradation. Most of these guidelines state that when biological samples are stored at room temperature, it is preferable to use paper containers (bags, boxes, envelopes) and avoid plastic packaging, since packaging biological samples in plastic can create favorable conditions for the growth of bacteria and mold, which could have detrimental effects on the sample [19,20]. However, the sample that was stored for 0.5 years had a higher DI than the sample stored for 2 years, even though the former had less storage time in a cardboard box, while the latter sample was stored for a longer time in a plastic folder. On the other hand, the two samples stored for 31 years (31a and 31b) showed very different DI values (276 vs. 2.4), even though both were stored in plastic containers (Table 1). Therefore, factors other than the storage container material may be more important in DNA degradation. For example, sample deterioration could be accelerated if it is not completely dry when packaged [21].

In umbilical cord samples with storage times between 0.5 and 21 years, DNA concentration values were highly variable (range 0.23–127.2 ng/µL) and almost always decreased and increased alternately between one time point and the next. The DI values of those samples also showed high variability (range 0.8–5.3) and, like the DNA concentrations, did not follow a linear pattern over time (Table 1). In all those samples, a complete profile was obtained that had exactly the same alleles as its reference sample.

Thus, all samples belonging to this storage time range had DNA concentrations > 0.01 ng/µL and, therefore, all were suitable for DNA typing [22]. In fact, a large amount of DNA was obtained from some of those samples (samples with 0.5, 12, and 21 years of storage, Table 1). These DNA extraction yields make the umbilical cord a good sample for direct comparison in forensic identification. Furthermore, as observed in this study, it is unlikely that mixed genetic profiles will be obtained from umbilical cords, something that occurs frequently with other types of samples [23]. We found no reports in the literature on the yield of DNA extraction from umbilical cords with different storage times to compare our results. Rajatileka and colleagues isolated DNA from 31 umbilical cord samples that had been stored for less than one month. On average, they obtained 117.3 ± 112.9 ng/µL of DNA [24]. Therefore, in that study a large variability in the amount of DNA obtained was also observed.

Regarding the DIs of these samples, it should be noted that, although only one of them presented a value lower than 1 (the one with 2 years of storage), all showed a complete profile with the same alleles as their corresponding reference sample. A DI < 1 indicates that DNA is not degraded, a DI between 1 and 10 indicates that DNA is slightly to moderately degraded, while a DI > 10 or blank (no value) indicates that DNA is significantly degraded [15]. However, although complete STR profiles were generated for all samples, signs of DNA degradation were observed, specifically the “ski slope effect,” which consists of a decrease in the peak heights of the longest loci on the electropherograms. Extreme DNA degradation can result in partial profiles with missing alleles at the longest loci (Figure 3, Ref. [25]).

Samples stored for 12 and 21 years showed high IPC CT values (32 and 34, respectively. Table 2). The presence of inhibitors in a sample reduces the success of the IPC amplification, with an increase in the IPC CT value. In general, IPC CT values above 30 are considered an indication of the presence of PCR inhibitors [15,26]. However, complete STR profiles were generated from these samples. Previous studies have shown that some samples can be amplified with varying degrees of success despite exhibiting some degree of PCR inhibition with high IPC CT values. This is because the GlobalFiler™ kit has proven to be much more tolerant to inhibitors than previous kits [15,27].

In the samples that had a longer storage time than those described above (with the exception of one of them), important changes were observed in the analyzed parameters (see sample with 26 years of storage and later, Table 1). DNA concentration values decreased and DIs values increased markedly, and as in the younger samples, both parameters did not follow a linear pattern over time. A complete STR profile was not observed in any of these samples (Figure 2), and the percentage of allele recovery decreased to 31% in the oldest umbilical cord analyzed.

IPC CT values were normal in these samples. However, samples with 31 (31a) and 47 years of storage showed a LA CT value of 36. According to the manufacturer of the DNA quantification kit, detection of CT values > 35 may indicate the presence of exceedingly low quantities of DNA [15]. Effectively, the DNA concentrations obtained from the samples with 31 (31a) and 47 years of storage were 0.08 and 0.05 ng/µL, respectively. In the sample with 26 years of storage, the DNA concentration obtained was so low (0.01 ng/µL), that not only was no LA CT value obtained due to the failed amplification of the large target in the quantitative PCR, but a high value (33) of the SA CT was also obtained (Table 2).

Taking into account only the analysis above, it could be considered that there is a cut-off in the storage period of the umbilical cord samples (21 years in our study) after which the analyzed parameters worsen considerably and incomplete STR profiles are obtained. However, from one of the samples that had been stored for 31 years (31b), a good concentration of DNA was obtained (12.0 ng/µL), along with a not very high DI of 2.4 and a complete STR profile with the same alleles as its reference sample (Table 1). In addition, all of their CT values were normal (Table 2). Thus, it is evident that in addition to storage time, other factors must influence the success of using umbilical cord DNA as a reference sample in forensic identification.

Most of the factors that could influence the quantity and quality of DNA obtained from umbilical cords could be related to their particular storage conditions. DNA is subject to degradation when stored at ambient conditions. The degree of DNA degradation can vary depending on several factors, including environmental conditions (light, humidity, temperature) and the presence of damaging agents (nucleases, oxidizing agents, bacterial growth), which cause hydrolytic and oxidative damage to DNA [28,29]. The samples we analyzed came from 15 different houses, so the exact storage conditions varied for each one, which could explain some of our results. However, our intention was not to test experimental conditions to find the parameters that would yield the best results from these samples, but rather to analyze cases that might arise in the actual practice of forensic analysis.

In order to facilitate the comparison of our findings with others previously published, in Table 3 we summarize the main characteristics of related works present in the literature.

Existing articles in the field are isolated cases or case series. In this work, we analyzed more samples and more STR loci, and we present the results of more parameters than in other studies. In all of those previous works, the positive identification of the problem samples was reported using umbilical cords as a reference. Of note, Hara et al. obtained a 15-loci STR profile from an umbilical cord stored for 77 years in Japan [9]. This finding could support the hypothesis regarding the importance of specific storage conditions for each sample in obtaining results useful for forensic identification.

Some limitations of this study must be mentioned. First, in some cases, there was little difference between the storage time of one sample and another. On the other hand, this fact revealed that there may be important differences in some of the analyzed parameters, regardless of storage time, which could depend on the specific conditions under which each sample was stored (see, for example, the DNA concentrations and DI values of the samples with 0.5 and 2 years of storage, Table 1). Second, the oldest umbilical cord we analyzed was 47 years old; we were unable to obtain an older one. However, we consider it feasible that, in actual forensic cases, the analyzed samples will be within the analyzed storage time range (from 0.5 to 47 years), so the results obtained are realistic. Third, the samples were analyzed only once. It would be interesting to perform duplicate or triplicate assays in a study devoted to determining the optimal parameters for analyzing this type of sample.

Finally, it has been established that parameters such as DNA quantity and DI, assessed together, could be used to predict the quality and usefulness of the expected STR profile, and to decide whether and how to process samples in order to maximize allele recovery [27,30]. In the samples with 26, 31 (31a) and 47 years of storage that we analyzed, low values of DNA concentration and high DI values were observed, and partial STR profiles were obtained (90, 47, and 31% of alleles recovered, respectively; Table 1). However, these partial profiles could still be used in forensic practice, even if only to rule out the identification of a sample, since in paternity and maternity tests the questioned sample must have at least one allele from each parent in all loci, and in direct identification tests, all alleles must match between the control sample and the questioned sample. Therefore, it is advisable not to rule out a priori the usefulness of genetic profiles from samples that present unfavorable values in the parameters obtained in tests prior to genotyping.

## 5. Conclusions

The results presented in this work indicate that storage time influences the quality of STR profiles obtained from umbilical cord samples, but that additional considerations, such as storage conditions, must be taken into account for the use of these samples in forensic identification.

## Figures and Tables

**Table 1:** DNA yield, storage container, degradation index (DI), and completeness of the genetic profile of the analyzed samples.

Sample Age (Years)	Storage Container	DNA Concentration (ng/µL)	DI	Profile Completeness (%)
0.5	Cardboard box	90.6	5.3	100
2	Plastic folder	0.40	0.8	100
4	Plastic box	1.93	1.7	100
6	Plastic box	1.24	1.8	100
7	Plastic box	9.21	1.7	100
9	Cardboard box	8.15	3.1	100
12	Cardboard box	127.2	4.4	100
15	Cardboard box	0.23	4.6	100
18	Plastic bag	13.7	3.5	100
19	Paper envelope	21.6	3.8	100
21	Cardboard box	68.7	4.7	100
26	Plastic folder	0.01	NR	90
31a	Plastic bag	0.08	276	47
31b	Plastic folder	12.0	2.4	100
47	Glass jar	0.05	275	31

Two samples of 31 years were analyzed (31a and 31b); NR: no result was obtained due to the failed amplification of the large target.

**Table 2:** Cycle thresholds of the small and large amplicons of DNA obtained from the umbilical cordon samples, and of the internal PCR control and the non-template control of the quantification assays.

Sample Age (Years)	SA CT	LA CT	IPC CT	NTC CT
0.5	21	21	30	29
2	28	26	30	29
4	25	23	30	29
6	26	24	30	29
7	23	21	30	29
9	24	24	29	28
12	20	19	**32**	29
15	28	28	29	29
18	23	23	29	28
19	22	21	30	29
21	21	20	**34**	29
26	**33**	**NR**	29	29
31a	30	**36**	29	29
31b	23	23	29	28
47	30	**36**	29	29

Two samples of 31 years were analyzed (31a and 31b); SA: small amplicon; LA: large amplicon; IPC: internal PCR control; NTC: non-template control; CT: cycle thresholds; NR: no result was obtained due to the failed amplification of the large target. Altered CT values are in bold.

**Table 3:** Main characteristics of related works previously published in the field.

Number of Samples Analyzed	Storage Time (Years)	Number of Autosomal STR Loci Analyzed	Type of Identification Test	Reported Results	Reference
1	~30	3	DC	STR typing	[6]
5	9 months 7 38 40 44	10 14 10 12 12	PT, MT PT PT, MT PT PT	PT, MT	[7]
2	<2	9	DC, MT	STR typing MT	[8]
1	77	15	MT	STR typing MT	[9]
1	44	15	PT	STR typing	[10]
15	0.5, 2, 4, 6, 7, 9, 12, 15, 18, 19, 21, 26, 31a, 31b, 47	21	DC	STR typing, DNA yield, DNA integrity, PCR inhibitors	This work

Two samples of 31 years were analyzed (31a and 31b); DC: direct comparison; PT: paternity test; MT: maternity test.

## Data Availability

The original contributions presented in the study are included in the article and Supplementary Materials, further inquiries can be directed to the corresponding author.

## Supplementary Materials

The following supporting information can be downloaded at: https://www.mdpi.com/article/10.3390/genes17091066/s1, Figure S1: Graph of DNA concentration versus sample age; Figure S2: Graph of degradation index versus sample age; Figure S3: Graph of profile completeness versus sample age; Table S1: Short tandem repeat results for samples that yielded an incomplete profile.

## Abbreviations

The following abbreviations are used in this manuscript:

bp	Base pairs
°C	Degrees Celsius
CT	Cycle threshold
DI	Degradation index
DNA	Deoxyribonucleic acid
DTT	Dithiothreitol
IPC	Internal PCR control
LA	Large amplicon
M	Molar concentration
µg	Micrograms
mg	Milligrams
mL	Milliliters
ng	Nanograms
NTC	Non-template control
PCR	Polymerase chain reaction
R^2^	Correlation coefficient
RFU	Relative fluorescence units
SA	Small amplicon
