# Supplementary material for: Effect of Storage Time on DNA Obtained from Umbilical Cord Used in Forensic Identification
Source: Genes (Basel). 2026 Sep 3;17(9):1066. doi: 10.3390/genes17091066 (PMC13606748; doi:10.3390/genes17091066)
Supplement: Supplementary file 1 [file genes-17-01066-s001.zip › Table S1.pdf]

Table S1. Short tandem repeat results for samples that yielded an incomplete profile.

	26 years		31 years (31a)		47 years	
**Locus**	**Saliva**	**UC**	**Saliva**	**UC**	**Saliva**	**UC**
D3S1358	15/18	15/18	15/17	-/17	13/17	13/17
vWA	16/17	16/17	16/16	16/-	14/17	-/-
D16S539	12/12	12/12	11/12	-/-	10/12	-/-
CSF1PO	8/10	8/-	11/12	-/-	11/12	-/-
TPOX	8/8	8/8	8/9	-/-	8/8	-/-
D8S1179	10/13	10/13	13/13	13/13	13/14	13/14
D21S11	29/29	29/29	30/31.2	-/-	30/30	-/-
D18S51	12/16	12/16	12/18	-/-	12/13	-/-
D2S441	11/11	11/11	10/11	10/11	10/11	10/11
D19S433	15/15.2	15/15.2	12/14	12/14	13/14.2	13/14.2
TH01	6/6	6/6	7/7	7/7	6/7	-/-
FGA	22/24	22/24	23/25	23/-	22/26	-/26
D22S1045	15/16	15/16	15/15	15/15	15/16	15/-
D5S818	11/11	11/11	11/13	11/13	11/13	-/-
D13S317	10/12	10/12	8/13	-/-	9/9	-/-
D7S820	10/12	10/-	8/11	-/-	10/10	-/-
SE33	26.2/28.2	26.2/28.2	15/30.2	-/-	17/18	-/-
D10S1248	16/16	16/16	13/14	13/14	14/16	14/16
D1S1656	16/17	16/17	13/16.3	13/16.3	12/17.3	-/-
D12S391	17/19	17/19	20/21	-/-	21/24	-/-
D2S1338	20/24	-/-	17/24	-/-	23/25	-/-
Amelogenin	XX	XX	XX	XX	XX	XX

UC: umbilical cord; -: no result was obtained.
